# Migraine Type-Dependent Patterns of Brain Activation After Facial and Intranasal Trigeminal Stimulation

**DOI:** 10.1007/s10548-022-00924-x

**Published:** 2022-11-12

**Authors:** Antje Haehner, Gudrun Gossrau, Franziska Bock, Thomas Hummel, Emilia Iannilli

**Affiliations:** 1 Smell & Taste Center, Deptartment of Otorhinolaryngology, TU Dresden, Dresden, Germany; 2 Headache Outpatient Clinic, University Pain Center, University Hospital, TU Dresden, Dresden, Germany; 3 Deptartment of Biomed Sci, State Univ of New York at Albany, Albany, NY USA; 4 Deptartment of Psychology, K-F University of Graz, Graz, Austria

**Keywords:** Headache, Clustering analysis, EEG, Pain, Odor, Nose

## Abstract

In migraine, the trigeminal nerve is intimately involved in the pathophysiology of the disease. We hypothesized that alterations in the sensory trigeminal activation in migraine would be reflected by EEG-derived event-related potentials (ERP). We aimed to investigate differences in the temporal and spatial processing of trigeminal stimuli between interictal migraine patients and healthy subjects. ERP to trigeminal stimuli were recorded at 128-channels to allow localization of their cortical sources with high temporal resolution. Seventeen patients with episodic migraine without aura, 17 subjects with episodic migraine with aura, and 17 healthy subjects participated in the study. The first branch of the trigeminal nerve was stimulated using intranasal chemical (CO_2_), cutaneous electrical, and cutaneous mechanical (air puff) stimuli. Analyses were performed with regard to micro-state segmentation, ERP source localization, and correlation with the patients’ clinical characteristics. Topographical assessments of EEG configurations were associated with the pathological condition. The source analysis revealed altered trigeminal-sensory response patterns in the precuneus, temporal pole, and cerebellum for both migraine groups during the interictal phase. The estimated current source density was positively correlated with migraine disease duration, indicating brain functional and structural changes as a consequence of the disease. Hyperactivity of the cerebellar posterior lobe was observed as a specific trigeminal response of migraine patients with aura. In conclusion, our results suggest the presence of brain changes accompanying the advancement of migraine as an expression of dysfunctional central pain processing. Hence, we identified EEG patterns in response to mechano-/chemosensory stimuli that can serve as biomarkers of migraine.

**Supplementary Information:**

The online version contains supplementary material available at 10.1007/s10548-022-00924-x.

## Introduction

The trigeminal nerve is intimately involved in the pathophysiology of migraine. While the ophthalmic and maxillary branches of the nerve are purely sensory, the mandibular branch has sensory and motor functions. A variety of sensory inputs from the scalp, face, nose, mouth, and meninges are processed by the trigeminal pathway providing tactile, proprioceptive, and nociceptive as well as chemosensory information. Nociceptive afferents in the nasal cavity have free nerve endings located within the lining of the nasal vestibule and nasal chambers. When stimulated by chemical substances, they lead to sensations like stinging, burning, warmth, or cold (Doty et al. 1978; Reeh and Kress 2001), which are significant components of the extensive somatosensory representation of the craniofacial region in the central nervous system.

The exact mechanisms underlying migraine pain are still not fully understood, mainly because of the absence of any identifiable brain pathology in established imaging and EEG techniques. It is assumed that migraine headaches originate from trigeminal nerve terminals in meninges due to neurogenic inflammation (Goadsby 2007; Messlinger et al. 2020; Moskowitz and Macfarlane 1993; Coppola et al. 2020).

Data on functional imaging show that the posterior part of the hypothalamus is significantly activated in the pre-ictal period of the migraine attack and a role its onset has been suggested (Schulte et al. 2017; Maniyar et al. 2014). Furthermore, changes in hypothalamic-brainstem connectivity are thought to drive the attacks (Schulte and May 2016). Observing spontaneous migraine attacks in a patient by magnetic resonance imaging revealed altered functional coupling between the hypothalamus and (a) spinal trigeminal nuclei and (b) the dorsal rostral pons pre-ictally. Hence, a top-down activation of trigeminal structures seems likely. As a result, peripheral and central trigeminal nerve endings release pro-nociceptive neurotransmitters, i.e., calcitonin gene-related peptide (CGRP) (Goadsby et al. 2017). Peripheral and central sensitization results in the transduction of nociceptive stimuli, which then may clinically manifest as migraine headache.

Although, the trigeminal system plays a major role in migraine pathophysiology, investigations of trigeminal sensory pathways in migraines using multichannel EEG are lacking. Previous experimental approaches relying on blink reflex (Sandrini et al. 2002; Brooks and Fragoso 2013) and/or classical pain-related event-related potential (ERP) recordings (Sohn et al. 2016; Valeriani et al. 2003) pointing towards abnormal temporal processing of external stimuli and dysfunctional sequential recruitment of neuronal networks involved in pain-processing in migraine, possibly the pathophysiological background of the disease (de Tommaso et al. 2014). Psychophysical tests in patients with migraine showed a mechanical hypersensitivity in trigeminal and non-trigeminal regions, most pronounced in relation to a migraine attack (Scholten-Peeters et al. 2020).

We hypothesized that alterations in the trigeminal activation in migraine would be reflected by EEG recordings based on a sufficient temporal and spatial resolution. The present study aimed to investigate whether there are differences in the processing of trigeminal stimuli between migraine patients with (PMA) and without aura (PM) and healthy subjects (N) expressed as different brain response patterns. Specifically, responses were studied during the interictal period with regard to three types of stimuli: (a) intranasal chemical nociceptive stimulation with gaseous CO2, (b) cutaneous electrical stimulation, and (c) cutaneous mechanical stimulation using air puffs. To determine whether there were differences in trigeminal pain-processing at different brain levels, we used the recording of ERPs obtained with 128 channel EEG. This allowed us to examine the spatial distribution of the ERP sources inside the cortices.

## Methods

### Participants

Fifty-one right-handed subjects participated in the study, including 17 PM patients (mean age ± sd = 40.1 ± 13.1 years), 17 PMA patients (mean age ± sd = 30.1 ± 10.3 years) and 17 N subjects (mean age ± sd = 37.8 ± 11.2 years). Patients were diagnosed according to the International Headache Society (IHS) criteria of the International Classification of Headache Disorders (Headache Classification Committee ICHD-III 2018) for “migraine without aura” and “typical aura with migraine headache.” Exclusion criteria included a previous history of olfactory disorders due to sino-nasal disease, head trauma, neuropsychiatric disease (except mild depression) and neurological disease (e.g., Parkinson’s disease, Alzheimer’s disease). The study protocol was approved by the Ethics Board of the Faculty of Medicine at the TU Dresden (protocol number EK 58022015). Detailed information about the experiment was given to all participants, and informed written consent was obtained. All aspects of the study were performed following the Declaration of Helsinki.

### Questionnaires

Migraine-related disability was assessed using the migraine disability score (MIDAS; sum score > 20 corresponds to high migraine-related disability) (Stewart et al. 2001) and the Headache Impact Test (HIT-6; sum score > 60 corresponds to high migraine-related disability) (Kosinski et al. 2003). To assess clinically relevant depression, patients completed the Beck Depression Inventory (Beck et al. 1996).

### Experimental Procedure

The study was divided into two sessions, separated by at least five days. During first visit, participants underwent olfactory and trigeminal testing and training for the experimental condition to familiarize them with the study procedures. Olfactory function was tested using the „Sniffin' Sticks” identification test (Hummel et al. 1997). Trigeminal testing was performed using a lateralization task (Frasnelli et al. 2009). Further, the Beck Depression Inventory-II (BDI) (Beck et al. 1996) was administered to assess self-reported symptoms of depression and to exclude moderate or severe depression in participants. Then eligible subjects were instructed through a training session simulating the actual experiment by randomly receiving a short trial of the three stimulus conditions. They also learned to perform a simple computer game based on tracking a white square with the mouse pointer, introduced to maintain stable concentration levels and avoid unwanted ocular movements that might produce EEG artifacts.

The EEG with 128 channels (Ag–AgCl active-electrodes, BioSemi, Amsterdam, NL) was recorded during the second visit. The first branch of the trigeminal nerve was stimulated in three different ways; described below. Electrodes were mounted on a standard 10/10 System head cap (BioSemi cap) and connected to the EEG amplifier (BioSemi Active Two AD-box). The outputs of all the AD converters were digitally multiplexed and sent via a single optical fiber to a USB2 Receiver (BioSemi) connected to a PC, where the data were registered and stored through acquisition software (BioSemi ActiveView 605). The sampling frequency of the signal was set at 512 Hz. An electrode gel (Signa gel—Parker laboratories, Inc. Fairfield, New Jersey, USA) was applied to each electrode tip to maximize the conductivity and reduce the impedance. Four pairs of flat-type, active electrodes were used to record the vertical electrooculogram (EOG), the horizontal EOG, both earlobes, and both mastoids. The olfactometer trigger was registered synchronously to the EEG signal. The whole acquisition lasted 120 min, with a break in between. The order of the conditions was randomized across the subjects. Acoustic white noise was played through headphones to cover the background noise. Participants were asked to rate intensity (0, not perceived to 100, extremely strong) and pleasantness (− 5, very unpleasant to + 5, very pleasant, 0 neutral) on a visual analog scale during the experiment.

### Stimulus Presentation

The three stimuli, intranasal nociceptive chemical, cutaneous electrical, and cutaneous mechanical (air-puff), were delivered to areas innervated by the first trigeminal branch. For chemical nociceptive stimulation, gaseous CO2 was chosen because it does not produce a concomitant odorous sensation which might interfere with pain sensation. CO2 was presented to the right nostril, while both electrical and mechanical stimuli were presented to the right forehead.

The chemical stimulation was presented using a computer-controlled air-dilution olfactometer (OM6B; Burghart, Wedel, Germany) with a constant flow of odorless, humidified air of controlled temperature (7.2 l/min with an 80% relative humidity and 36 °C). The electrical stimulus (500 µs duration, 200Vmax) was delivered through Ag–AgCl electrodes (COP10S-SEI; EMG s.r.l., Cittadella, Italy) using a constant current stimulator (Digitimer DS3; Digitimer Ltd., Welwyn Garden City, Hertfordshire, UK). The electrode was attached to the skin on the right eyebrow, innervated by the trigeminal nerve’s ophthalmic branch, with a ground electrode on the left forearm. However, due to the presence of large EEG artifacts, the electrical condition was excluded from the EEG analysis. The mechanical stimulus was an air puff of 250 ms duration, generated by the olfactometer. The opening of the Teflon tubing, through which the puff was delivered, had a diameter of 2 mm; the stimulator outlet was positioned approximately at 5 mm distance from the skin surface pointing towards the right forehead, airflow was 7.2 l/min. This stimulus was irritating but not painful.

### Stimulation Parameters

The setup of the stimulus condition was based on a pilot study aiming to match the stimuli in intensity. For this, 15 individuals were exposed to chemical, electrical, and mechanical conditions. They were asked to evaluate the conditions on a scale from 0 (not perceived) to 100 (extremely strong) in intensity. Initially, the chemical condition were split into two levels; CO2_low (44% v/v CO2) and CO2_high (56% v/v CO2). As regards the electrical stimulation, we first set the electrical threshold (minimum level of electrical stimulus detection), then increased the relative experimental condition by 20% of this value. The mean ratings and standard deviation for the group in each condition are reported in supplemental digital content- (SDC-)Table 1.

**Table 1:** Clinical and demographic data of episodic migraine patients with aura (PMA)

Age	Sex	BDI	Aura	Midas	HIT-6	Years with migraine	Migraine days last 3 month
32	F	37	y	23	63		
18	F	24	Seeing stars, silverish flicker scotoma central, concentric visual field narrowing, hypaesthesia arm, leg, aphasia	46	66	10	24
28	F	24	Flickery lights temporal, dysesthesia arm	160	65	5	75
58	F	18	y	112	55	38	21
51	F	24	Flicker scotoma temporal	71	63	13	15
24	F	1	y	1	69		
27	F	2	Dysesthesia arm, leg	21	54	5	12
28	f	1	y	7	57		
39	F	16	y	12	62		
31	F	12	y	160	65		
26	M	1	y	3	53		
26	F	5	y	3	55		
22	F	5	y	8	53		
24	F	1	y	5	54		
26	F	0	y	5	56		
27	M	7	y	19	63		
25	F	9	y	50	64		

*y* yes

A Friedman test revealed a statistically significant difference across the 4 intensity ratings [χ^2^(3, n = 15) = 20.3, p < 0.001]. Higher median (Md) intensity values were observed for CO2_high (Md = 52) compared to CO2_low (Md = 31), electrical condition (Md = 32), and mechanical condition (Md = 33). A posthoc test (Wilcoxon) with Bonferroni correction showed no significant differences between CO2_low vs. electrical condition nor for CO2_low vs. mechanical or electrical condition vs. Puff (p > 0.16).

Therefore, as the final set of experimental conditions, we chose (1) cutaneous mechanical stimulation using air puffs, (2) cutaneous electrical stimulation (20% higher than the individual threshold), (3) intranasal chemical stimulation with gaseous CO2 (44% v/v CO2). The three stimuli were presented at intervals of 20 s (range 18–22 s) with 50 repetitions each.

### Data Analysis

ERP were evaluated using the Cartool software (Brunet et al. 2011). The pre-processing steps included filtering (low pass 15 Hz, high pass 0.1 Hz, and notch 50 Hz), baseline correction, and manual artefact rejection. For each subject, a grand average (GA) was computed for each condition. The single-subject GA were entered in the group grand mean (GM) for a final total of 9 GM (3 groups × 3 conditions).

Functional microstates, as defined by Lehmann and colleagues (Lehmann et al. 1987), were computed simultaneously for the three groups in each condition using a microstate segmentation algorithm (Katayama et al. 2007; Pascual-Marqui et al. 1995). Topographical atomize and agglomerative hierarchical clustering (T-AAHC) analysis was used to identify the stable map voltage topography (microstates). The best clustering was individuated by maximizing a meta-criterion, based on the median of all optimal numbers of clusters defined by 6 criteria as described in (Brechet et al. 2019). Cluster maps correlating more than 80% were merged, and segments less or equal to 24 ms were rejected. The synthetic maps were then visually compared to the time course topographical maps obtained by the grand mean for each condition Figs. 2i or 5i. Segmentation of the GM can identify similarities and differences in the time course of the post-stimulus brain process. The identified micro-states, a set of template maps that best represent a certain epoch segment, were fitted back to the single subject GA data using a procedure based on a spatial correlation of the cluster maps (Michel 1999). Ultimately, the fitting procedure generated parameters of interest in the relevant maps that pinpoint specific brain process characteristics to the group/condition. The corresponding anatomical brain structures responsible for the voltage scalp map distribution were identified using source localization techniques based on autoregressive average (LAURA) algorithm (Grave 2004) using 6000 source points distributed in the gray matter and integrated into Cartool software (Brunet et al. 2011). The inverse solution matrix was multiplied by the GA of each subject to obtain the results of the inverse solution, which represents the estimated current source density (eCSD). Coordinates of the localized sources are reported in MNI- (Montreal Neurological Institute) space. The analysis of psychophysical, track performance, and demographical data was carried out using IBM SPSS Statistics v.23 (SPSS Inc., Chicago, Ill., USA).

The eCSD of critical brain activities for each subject was extracted in a spatial region of interest (sROI) defined by the solution point in the maximum activity and the six cartesian nearest neighbors and mediated in the timeframe defined by the fitting back procedure.

The following paragraph describes our hypothesis and the relative statistical tests used to verify them. First, we used a Chi-square test to test the gender and age homogeneity hypothesis. To test the hypothesis of differences among the three patient groups in Odor Identification (OI), trigeminal lateralization (TL), and BDI, we will apply the non-parametric ANCOVA test (Quade 1967) ref. Quade’s test[IE1]). The same test will be applied to compare psychophysics and performance within and between the groups. The preventive medication will be set as co-variate. Successively, for each variable/condition extracted by the microstates fitting back, we will test the differences among the groups using a Kruskal–Wallis one-way analysis of variance. Finally, we will measure the correlation between eCSD and clinical data using Spearman rho correlation.

## Results

### Demographics and Migraine Screening

The three groups were homogenous in gender [χ^2^(2, n = 51) = 0.30, p = 0.86, effect size (V) = 0.01], and age [χ^2^(2, n = 51) = 4.47, p = 0.11, effect size (V) = 0.02] with a relative mean age ± s.d. N = 38 ± 11, PM = 40 ± 13, PMA = 30 ± 10.

Of 17 patients with migraine without aura, 5 showed pronounced migraine prodromes, 4 were on prophylactic migraine medication, and 9 on nonmedication prophylactic therapy (Table 3). In addition, 6 of the patients complained about tension type headache or facial pain, and 8 patients about pain in other body regions. 11 patients took acute migraine medication and 6 consumed analgesics for pain conditions other than migraine. A psychological diagnosis was seen in 3; concomitant somatic diseases in 4 patients. Of 17 patients with migraine with aura, 2 showed pronounced migraine prodromes, only one was on prophylactic migraine medication and 2 on non-medication prophylactic therapy. Four of the patients complained about tension type headache or facial pain, and 3 patients about pain in other body regions. Six patients were on prescribed acute migraine medication, and 1 consumed analgesic for pain conditions other than migraine. A psychological diagnosis was seen in 4, and concomitant somatic diseases in 4 patients. In each patient group, 8/17 (47%) presented a Migraine Disability Index (MIDAS) of ≥ 21 points, expressing a rather strong negative impact of migraine on daily life activities. Headache Impact Test (HIT-6) value of ≥ 60 points corresponding to the severe migraine-related burden on daily life was seen in 7/17 (41%) and 9/17 (53%) of patients with migraine without aura and migraine with aura, respectively. For further details, see Tables 1 and 2.

**Table 2:** Clinical and demographic data with Episodic migraine Patients without aura (PM)

Age	Sex	BDI	Midas	HIT-6	Years with migraine	Migraine days last 3 month
26	F	3	12	64	10	15
43	F	13	70	61	20	6
50	F	13	85	53	30	45
54	F	2	5	54	30	5
57	F	4	94	67	35	35
23	F	4	2	65		
21	F	9	25	59		
26	M	0	2	50		
55	F	8	24	51	43	6
55	F	6	45	57	30	27
44	M	14	6	62		
50	F	5	12	51		
24	M	4	14	56		
46	F	1	30	51	37	24
40	F	13	9	49	20	15
43	F	16	19	66	19	30
25	F	14	10	73		

Non-parametric ANCOVA test between groups

**Fig. 1:**
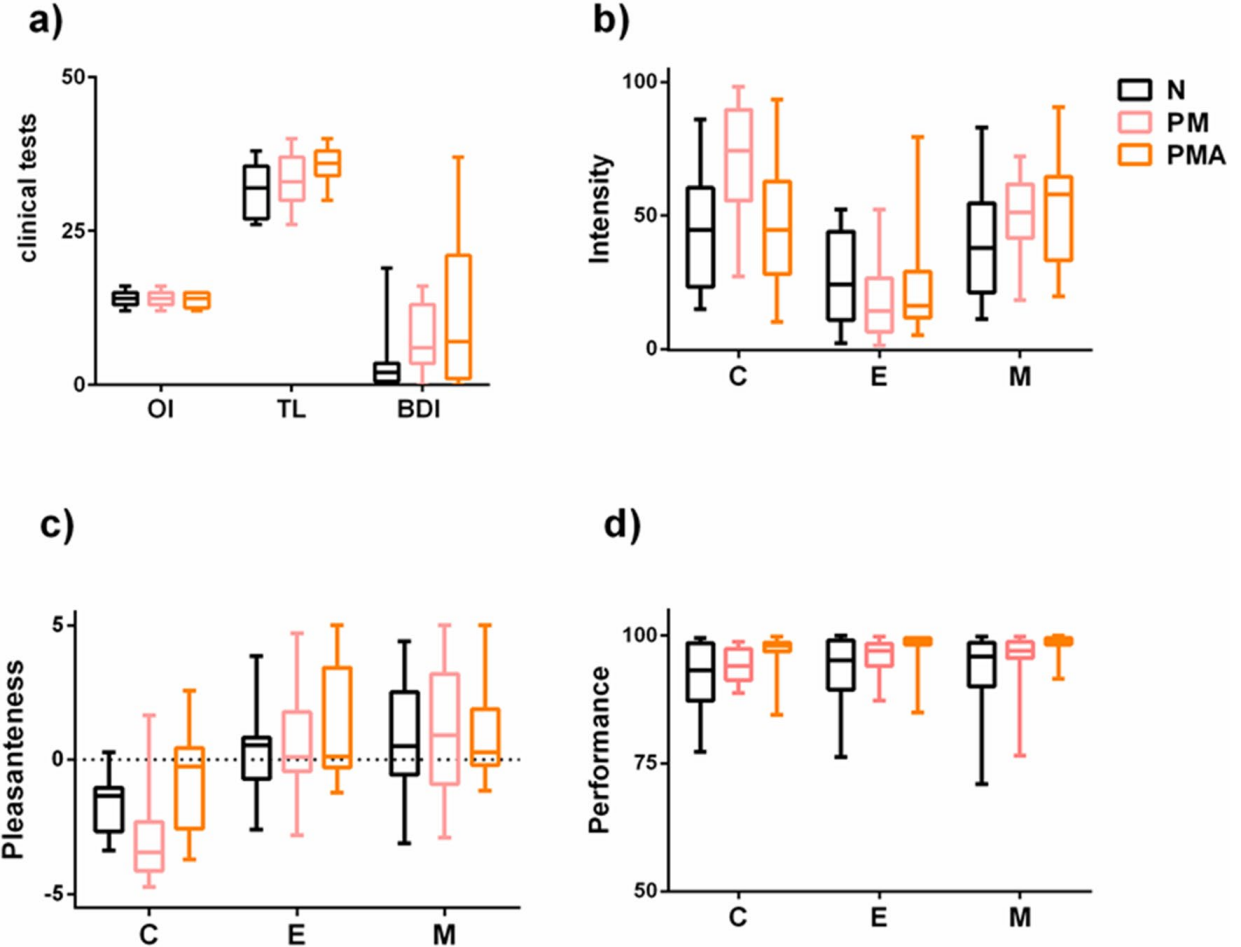
Boxplots of the clinical tests (**a**), olfactory identification (OI), trigeminal lateralization (TL), and BDI, obtained during the participant screening, and of the psychophysical tests, (**b**), intensity, (**c**) pleasantness, and (**d**) performance during the experimental session in response to the three stimulus conditions, electrical (E), chemical (C) and mechanical (M), for the three groups in study N, PM, and PMA. Values expressed by the box plot are 1-to-99 percentile and median

A non-parametric ANCOVA test with the dependent screening variables of odor identification (OI), trigeminal lateralization (TL), and BDI, with group as a categorical variable and the preventive medication used as co-variate. The test revealed no statistically significant differences for odor identification, but for trigeminal lateralization [F(2,48) = 5.101, p = 0.010, effect size (r) = 0.38and for the BDI (F(2,48) = 4.142, p = 0.022, effect size (r) = 0.33 (strong)]. BDI post-hoc analyses (Tukey) showed significant differences between N- and PMA- group (p = 0.032, effect size (d) = 0.53 (medium), mean difference (N-PMA) = − 12.41, CI(95%) = (− 23.92,− .90) Trigeminal lateralization test post-hoc analyses (Tukey) revealed significant differences between N- and PMA- groups [p = 0.00.13, effect size (d) = 0.54 (strong), mean difference (N-PMA) = − 13.82, CI(95%) = (− 25.14, − 2.) and between PMA and PM (p = 0.038, effect size (d) = 0.54 (strong), mean difference(PMA-PM) = 11.84, CI(95%) = (0.52, 23.15)]. Data are illustrated in the boxplot in Fig. 1a.

### Intensity-, Pleasantness-, and Task-Performance Ratings During the EEG Session

The data are illustrated in Fig. 1b, c, and d.

### Psychophysics and Performance Statistical Results Within the Group

Statistical analysis revealed that the *N group* rated intensity [χ^2^(2, n = 15) = 6.15, p < 0.046] and pleasantness [χ^2^(2, n = 15) = 10.5, p < 0.005] differently among the three stimulus conditions. The post-hoc analysis revealed that N rated the intensity of the CO2 higher than the intensity of electric (mean difference intensity (C-E) = 16.95, CI(95%) = (3.49, 30.40), p < 0.01, effect size (r) = 0.429) and less pleasant than both electric and the air-puff (mean difference hedonics (C-E) = − 3.59, CI(95%) = (− 4.01, − 9.63), p < 0.05 effect size (r) = 0.375; mean difference hedonics (C-M) = − 3.92 CI(95%) = (− 2.31, − 0.44), p < 0.01, effect size (r) = 0.449).

The *PM group* presented with significantly different ratings between the three conditions both in intensity F{2, 47} = 38.604, p = 0.000 and hedonics F{2, 47} = 18.176, p = 0.000, where post-hoc analysis revealed that PM rated the CO2 and the air puff higher than electrical in intensity [respectively: mean difference intensity (C-E) = 58.32, CI(95%) = (16.74, 45.23), p < 0.005, effect size (r) = 0.532 and mean difference intensity (M-E) = 35.10, CI(95%) = (13.60, 31.73), p < 0.005, effect size (r) = 0.504] and CO2 pleasantness was lower than electrical and air puff [respectively: mean difference hedonics (C-E) = − 4.43, CI(95%) = (− 4.87, − 0.03), p < 0.005, effect size (r) = 0.567; mean difference hedonics (C-M) = ( − 4.73, − 0.07), p < 0.005, effect size (r) = 0.487].

The *PMA-group* presented significantly different ratings of intensity F{2,44} = 9.485, p = 0.003 (Fig. 1b, c). Post-hoc analysis showed that the electrical stimulus intensity was rated lower than the CO2 and the air puff [mean difference intensity (C-E) = 18.04, CI(95%) = (3.22, 33.77), p < 0.05, effect size (r) = 0.376; mean difference intensity(M-E) = 45.21, CI(95%) = (20.34, 64.21), p < 0.01]. All post-hoc tests were carried out with Wilcoxon with Bonferroni threshold correction. No statistically significant difference was detected for mean performance between stimulus conditions within the group (Fig. 1d).

### Psychophysics and Performance Statistical Results Between Groups

Non-parametric ANCOVA test on the categorical variable group (N, PM, PMA) and the dependent variables of electrical threshold stimulus acquisition, mean intensity (CO2/electrical stimulus/puff), mean hedonics (CO2/electrical stimulus/puff), and mean performance (CO2/electrical stimulus/puff) was performed using as co.-variate the preventive medications. Results with a medium effect size revealed a significant increase of the Chemosensory stimulation perception for PM versus both N and PMA groups, while the PMA group significantly prefer in comparison with PM the chemosensory stimulation. Finally, the PMA group was a better performer of the N group in all three conditions, in addition performed also better that PM during the CO2 conditions. A detailed account of all the statistics results is reported in Table 4.

**Table 3:** Results of a non-parametric ANCOVA test between groups that uses as co-variate the preventive medication

Condition	Variable				
	F{2,48}(p)	Mean difference (p)			Effect size (r)
		N-PM (p) CI(95%)	N-PMA (p) CI(95%)	PM-PMA (p) CI(95%)	
*Intensity*					
CO2	4.653 (.014)*	− 12.10 (.029)* (− 23.15, − 1.03)	− 0.07 (1.000) (− 11.13,11.00)	12.03 (.030)* (.96, 23.09)	0.36
Electric stimulus	.476 (.625)	4.79 (.596) (− 7.11, 16.69)	2.68 (.868) (− 9.70, 14.85)	− 2.21 (.898) (− 14.31, 9.89)	0.15
Air-puff	2.133(.131)	− 8.18 (.205) (− 19.61, 3.26)	− 8.77 (.172) (− 20.41, 2.85)	− .60 (.991) (− 12.24, 20.41)	0.22
*Hedonics*					
CO2	5.975 (.005)***	6.77 (.310) (− 4.28, 17.82)	− 8.32 (.175) (− 19.37, 2.73)	− 15.09 (.005)*** (− 26.14, − 4.05)	0.41
Electric stimulus	.247 (.782)	.913 (.983) (− 11.63, 13.46)	− 2.54 (.885) (− 15.48, 10.41)	− 3.45 (.792) (− 16.20, 9.30)	0.18
Air-puff	.056 (.945)	1.46 (.956) (− 10.88, 13.80)	1.38 (.962) (− 11.16, 13.93)	− .08 (1.000) (− 12.63, 12.47)	0.21
*Performance*					
CO2	5.118 (.010)**	.01 (1.000) (− 11.36, 11.38)	− 13.02 (.021)* (− 24.39, − 1.65)	− 13.03 (.021)* (− 24.40, − 1.66)	0.37
Electric stimulus	4.013 (.025)*	− 1.18 (.967) (− 12.75, 10.40)	− 12.14 (.046)* (− 24.08, − .20)	10.96 (.073) (− .81, 10.40)	0.34
Air-puff	4.132 (.023)*	− 4.35 (.633) (− 15.83, 7.12)	− 13.04 (.025)* (− 24.71, − 1.37)	− 8.69 (.181) (− 20.35, 2.98)	0.35
Electric threshold	.879 (.422)	6.29 (.423) (− 5.77, 18.36)	4.90 (.592) (− 7.16, 16.96)	− 1.40 (.958) (− 13.46, 10.66)	0.07

F{df1, df2}(p): ACOVA test results and relative p value; Mean difference and post hoc analysis (Tukey/Scheffé) with relative p value; CI(95%): 95% confidence interval. Effect size range: .1 < r < .3 small, 0.3 < r < 0.5 medium, r > .5 strong (Rea and Parker 1992)

*p < .05; **p < .01; ***p < .005

**Table 4:** Statistical test results for the variables extracted after the fitting back of the segmentation to the single subject grand average for the mechanosensory condition (air-puff)

time frame (tf)	Map	Mean duration	GEV	First onset	Max GFP
		p (H)			
1	5	0.1810 (3.419)	0.1738 (3.500)	0.3455 (2.125)	n\a
	6	0.7565 (0.5581)	0.806 (0.4314)	0.1126 (4.369)	﻿n\a
	9	0.0033 (11.40)**	0.0036 (11.28)**	0.2441 (2.820)	0.0199 (7.836)**
2	6	0.975 (0.05070)	0.7132 (0.6760)	﻿n\a	﻿n\a
	7	0.0471 (6.110)*	0.0225 (7.591)**	﻿n\a	﻿n\a
	8	0.9988 (0.002374)	0.9852 (0.02975)	﻿n\a	﻿n\a
	10	0.3467 (2.119)	0.3898 (1.884)	﻿n\a	﻿n\a
	12	0.0596 (5.639)^§^	0.0982 (4.641)	﻿n\a	﻿n\a
3	9	0.9402 (0.1233)	0.9071 (0.1951)	0.5935 (1.044)	0.3902 (1.882)
4	12	0.0051 (10.56)**	0.0485 (6.054)*	﻿n\a	﻿n\a
	**13**	0.0438 (6.256)*	0.0283 (7.131)*	﻿n\a	﻿n\a

	N	PM	PMA	Effect size (r)
Map9 (tf1)				
Mean duration CI(95%)	43.79 (16.08, 71.45)	38.33 (14.48, 62.17)	116.0 (63.56, 168.5)	0.49
GEV CI(95%)	0.144 (0.043, 0.246)	0.09 (0.02, 0.16)	0.28 (0.18, 0.31)	0.41
Max GFP CI(95%)	1.13 (0.59, 1.68)	1.23 (0.80, 1.66)	1.95 (1.60, 2.3=)	0.37
Map7 (tf2)				
Mean duration CI(95%)	7.64 (− 0.14, 15.42)	0.0002 (− 9e–005, 0.0005)	17.82 (− 0.08, 35.72)	0.32
GEV CI(95%)	0.006 (− 0.001, 0.012)	0	0.045 (− 0.005, 0.096)	0.40
Map12 (tf4)				
Mean duration CI(95%)	7.26 (1.69, 12.82)	23.44 (15.36, 31,52)	15.33 (8.06, 22.59)	0.39
GEV CI(95%)	0.014 (− 0.001, 0.030)	0.045 (0.020, 0.070)	0.023 (0.001, 0.044)	0.31
Map13 (tf4)				
Mean duration CI(95%)	27.92 (18.22, 37.62)	11.28 (2.42, 20.14)	16.24 (2.47, 30.00)	0.31
GEV CI(95%)	0.034 (0.001, 0.068)	0.005 (0.001, 0.009)	0.012 (− 0.001, 0.025)	0.37

p, p-value of the statistical test, H, the value of the Kruskal–Wallis test. In addition, mean values, 95% confidence intervals [CI(95%)], and effect size of the variable with statistically significant results are reported in detail

**p < 0.005; *p < 0.05

§Indicates a value at the threshold supposes a trend of significance; ﻿n\a= not applicable

## EEG Results

### Grand Average

A grand average (GM) for each group and condition was obtained on an epoched time range of 1.2 s; the relative figure is reported in the Supplementary Information (SI-Fig. 1).

**Fig. 2:**
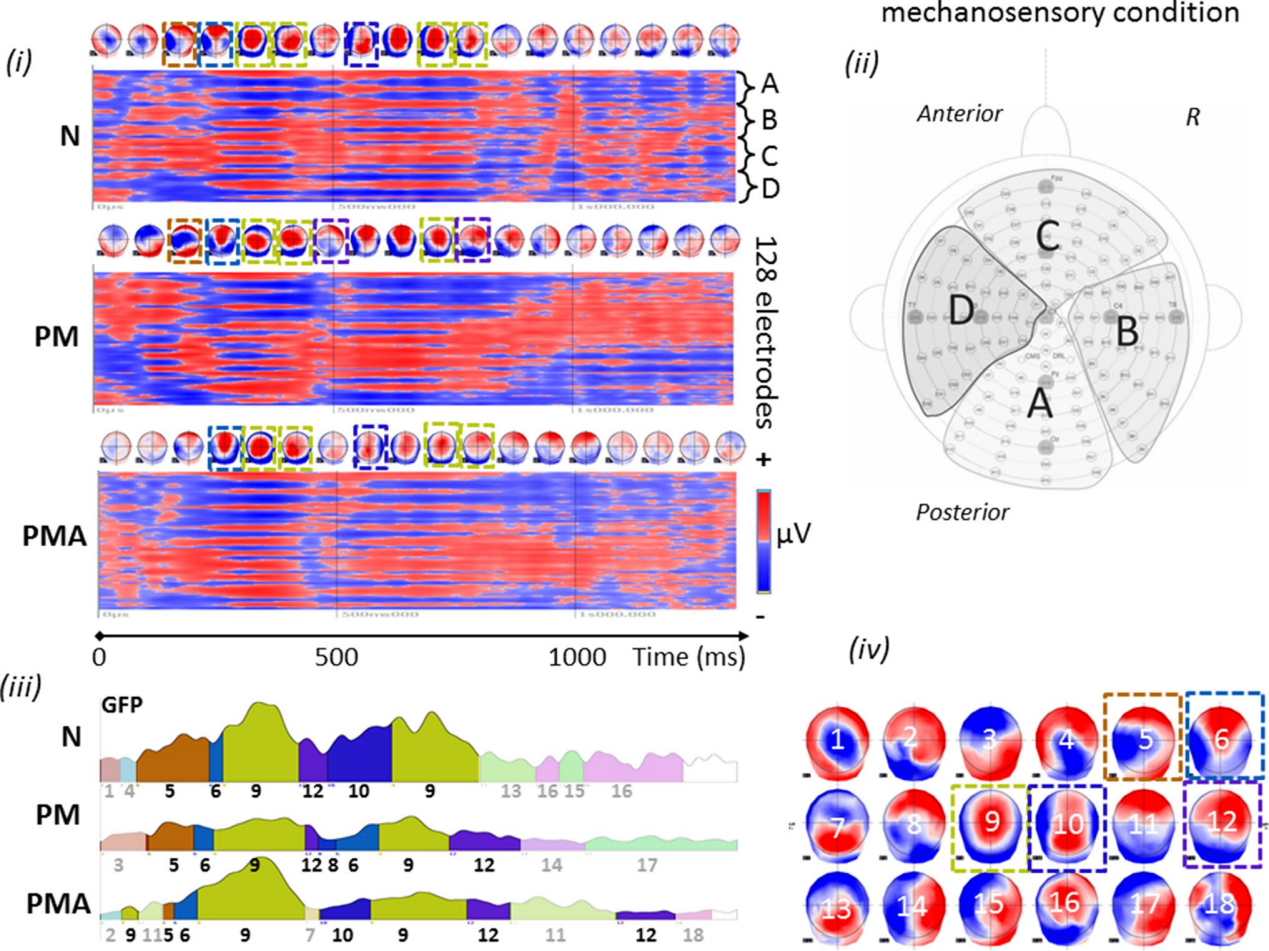
Identification of scalp predominant topography in the mechanosensory condition for the three groups in the study. **(i)** Grand mean for control (N), patients with migraine without aura (PM), and patients with migraine with aura (PMA) reported in red (positive)/blue(negative) voltage diagram, and relative topography averaged over 60 ms. Each diagram shows the voltage over the 128 channels (electrodes) used to register the scalp electroencephalogram. The electrodes were subdivided into four regions, indicated with A, B, C and D, each conveying 32 channels. **(ii)** Scalp distribution of the Biosemi 128-channel on the cap. A: central-occipital area, B: right centro-occipital area, C: Fronto-central area, D: left centro-occipital area. **(iii)** Microstates segmentation of the Grand Mean for the three groups in the study, N, PM, and PMA. The segments are projected on the global field power (GFP) of the relative GM, and they are reported color-coded and numbered in order of appearance. **(iv)** Microstates map topographies as defined by the segmentation process. A total of 18 maps describe the mechanosensory process across 1.2 s of post-stimulus onset for the three groups. The common maps across the groups are highlighted with dashed squares color-coded with the relative segment. The defined microstates are coherently found in the original topography as shown in **(i)**. Similar topographies are highlighted with dashed squares also color-coded with the relative microstates

**Fig. 3:**
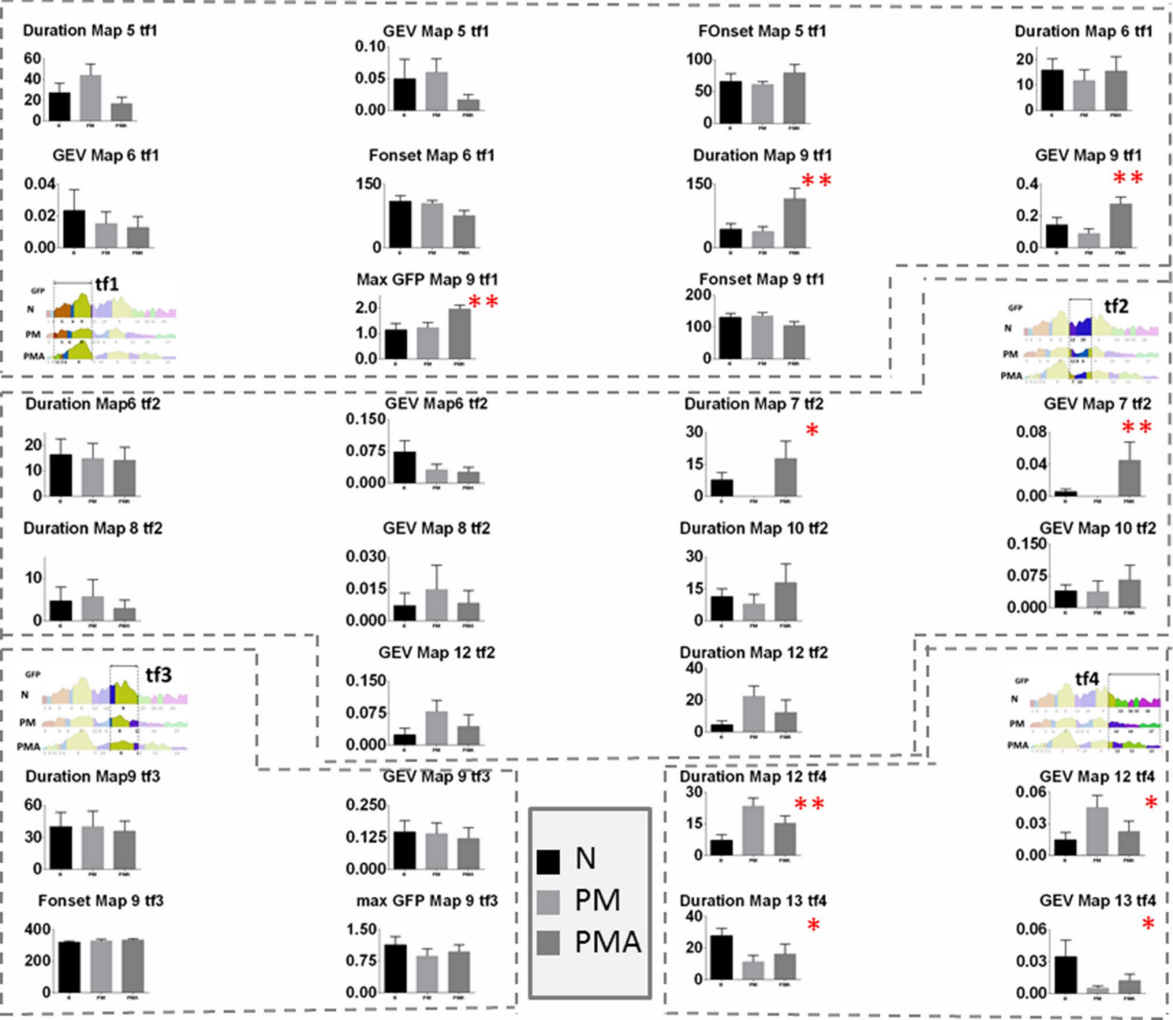
Mean and SE of the variables mean duration (Duration), global explained variance (GEV) and first onset (Fonset) of map 5, 6, 7, 8, 9, 10, 12, 13 in the time frame (tf) of interest. Statistically significant differences were found for duration, GEV and Max GFP of map 9 in tf1. Duration and GEV of map 7 in tf2. Duration and GEV of map 12 and 13 in tf4. The figure reports also the illustration of the relative tf within the epoch under analysis. tf1: 76 to 428 ms, tf2: 416 to 609 ms, tf3: 566 to 793 ms, and tf4: 729 to 1200 ms

The GM for each condition/group is also illustrated as red (positive)—blue (negative) voltage topography in Fig. 2i for PUFF and Fig. 3i for CO2. The corresponding scalp topographies, over 60 ms time average, are reported on top of each diagram and illustrate the brain activity time course of the 1.2 s post-stimulus onset.

### Microstate Segmentation and Source Analysis

#### Mechanosensory Condition

The segmentation, obtained from the 128-channel grand averages of the three groups, identified a total of 18 microstates, explaining 81.9% of the global variance [Fig. 2(iii), (iv)]. Identified stable topographies (microstates) were labelled with numbers from 1 to 18. For the control group (N) ten micro-states (namely 1, 4, 5, 6, 9, 12, 10, 13, 15, 16) represented the processing of the mechanosensory stimulus after the onset. For the PM group the epoch was segmented in eight micro-states, namely 3, 5, 6, 9, 12, 8, 14, 17 and for the PMA group in nine micro-states, namely 2, 9, 11, 5, 6, 7, 10, 12, 18. Microstates 5, 6 and 9 are common to the three groups within the same time frame of appearance (tf1: from 78 to 412 ms and tf2: from c.a. 572 ms to 792 ms) and same order (respectively in tf1 5, 6, and 9 and in tf2 9). Groups N and PM share map 12 from c.a. 417 ms to 472 ms (tf3), while N and PMA share map 10 from ca. 466 ms to 607 ms (tf4). Finally, PM and PMA share map 12 from c.a.732 ms to 880 ms (tf5). Interestingly, the initial time range of the epoch, from 0 to 123 ms, and the final part of the epoch, from 466 ms till the end, are represented by different microstates across groups suggesting distinct stimulus processing in the three groups. Comparing to the original data, microstate analysis clearly reflects it (Fig. 2I) with the identified microstates [Fig. 2(iii), (iv)]. Topographies in Fig. 2i similar to the microstates in Fig. 2(iii) and (iv) were highlighted with the same color frame.

##### Fitting Back to the Single Subject EEG-Grand Average

The fitted back procedure was based on the microstates included in the epoch segment that contained shared microstates. This corresponds to our segmentation of the mechanosensory condition to a time frame from 76 to 200 ms and includes microstates 5, 6, 7, 8, 9, 10, 12, and 13. For them, we extracted the variables ‘mean segment duration’ (Duration) and ‘global explained variance’ (GEV) for each subject. Both are indicative of how much the corresponding microstate is present in the relative single subject epoch segment. In addition, we also extracted the ‘first onset’ (Fonset) of appearance as an effect of the disease condition (map 5, 6, 9) and the maximum of the global field power (max GFP) in map 9. Based on the segmentation on the GM, we have defined time frames (tf) of interest where to extract the variable at the single-subject level, these were tf1, from 76 to 428 ms, for Duration and GEV, and Fonset of map 5, 6, and 9. In addition, in this time frame, we also extracted the maximal Global Field Power (max GFP) that reflects the strength of the signal for the specific condition. In tf2, from 416 to 609 ms, we extracted Duration and GEV of maps 6, 7, 8, 10, and 12. In tf3, from 566 to 793 ms Duration, GEV, first onset, and max GFP on the second appearance of map 9 and in tf4, from 729 to 1200 ms Duration, GEV of the second appearance of map 12, and the first appearance of map 13 were extracted.

A non-parametric Kruskal–Wallis one-way analysis of variance (one variable × 3 groups) was applied on the variable extracted from the fit back on the single subject above specified. The detailed statistical results are reported in Table 4. Mean values and SE of the variables are illustrated in Fig. 3 along with the time frame (tf) in the analysis.

Results showed that map 9 in tf1 had a significant different mean duration, GEV, and max GFP across the groups. Post hoc tests revealed that Duration, GEV, and max GFP were significantly bigger for the PMA condition with respect to N and PM.

Map 7 within tf2 had a significantly longer duration and bigger GEV in the PMA group than N and PM groups.

Finally, map 12 was significantly longer and explained more variance for both group PM and PMA with respect to N, and map 13 had a longer duration and a bigger GEV for N with respect to PM and PMA.

These results suggested that a longer duration of map 9 and map 7 and higher signal strength for map 9 are specifically observed in PMA. Map 12 is a unique topography associated with the migraine condition in both aura and non-aura symptoms. Finally, map 13 represents an exclusive topography characteristic of the normal condition.

*Source analysis for the mechanosensory condition.* The source analysis of map 9 revealed maximal activity in the right cerebellum, lower activity in the left cerebellum, and the left temporal inferior and superior pole.

The source analysis of map 7 pointed to a maximum activity in the left and right Cerebellum and the left and right gyrus rectus.

**Fig. 4:**
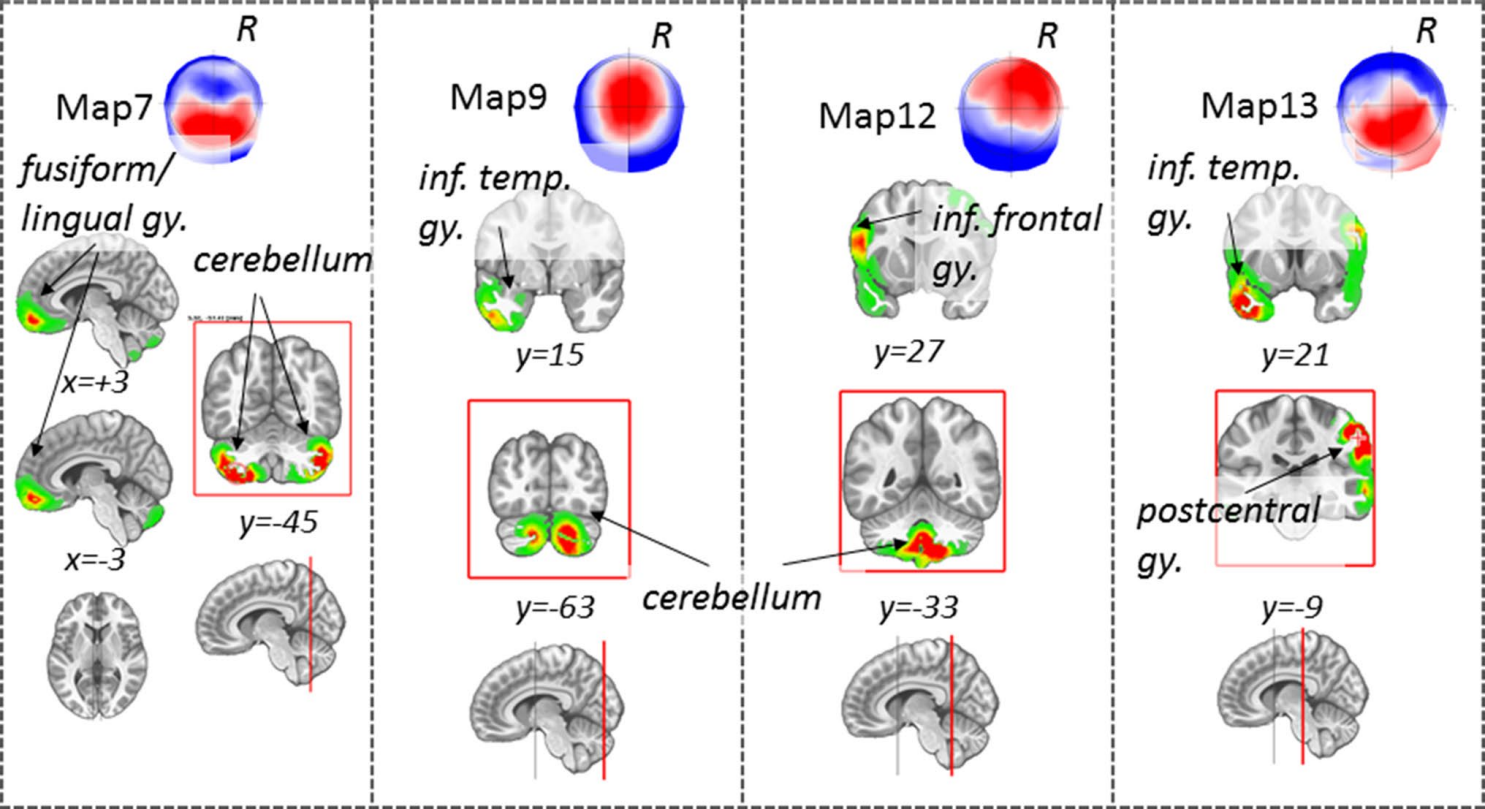
Source localization of the microstate of interest, map 7, 9, 12, and 13 within the mechanosensory condition. Maximal activities are highlighted within a red frame. R = right, coordinates are reported in MNI standard space

Map 12 is generated by a source in the right cerebellum and the left inferior frontal triangular area. Finally, map 13 is generated by a source in the right postcentral gyrus and left temporal pole. Brain activities associated with the discussed maps are illustrated in Fig. 4.

#### Chemosensory Condition

**Fig. 5:**
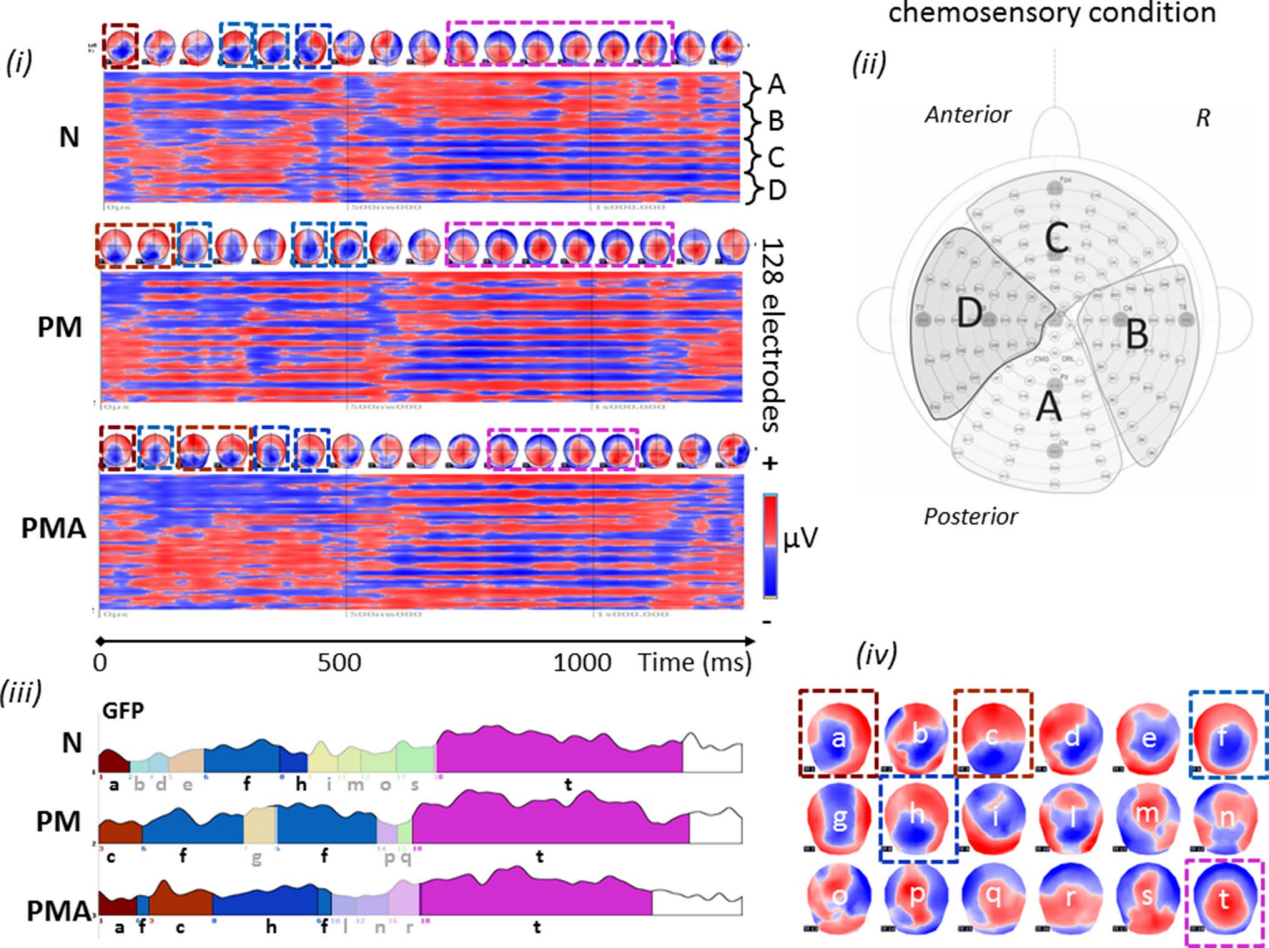
Identification of scalp predominant topography in the chemosensory condition for the three groups in the study. **(i)** Grand mean for control (N), patients with migraine without aura (PM), and patients with migraine with aura (PMA) reported in red (positive)/blue(negative) voltage diagram, and relative topography averaged over 60 ms. Each diagram shows the voltage over the 128 channels (electrodes) used to register the scalp electroencephalogram. The electrodes were subdivided into four regions, each conveying 32 channels. **(ii)** Scalp distribution of the Biosemi 128-channel on the cap. A: central-occipital area, B: right centro-occipital area, C: Fronto-central area, D: left centro-occipital area. **(iii)** Microstates segmentation of the Grand Mean for the three groups in the study, N, PM, and PMA. The segments are projected on the global field power (GFP) of the relative GM, and they are reported color-coded and numbered in order of appearance. **(iv)** Microstates map topographies as defined by the segmentation process. A total of 18 maps describes the chemosensory process across 1.2 s of post-stimulus onset for the three groups. The microstates, originally named with numbers in crescent order, were renamed with alphabetic letters from a to t, to avoid confusion with the maps identified in the mechanosensory segmentation. The common maps across the groups are highlighted with dashed squares color-coded with the relative segment. The defined microstates are coherently found in the original topography as shown in **(i)**. Similar topographies are highlighted with dashed squares also color-coded with the relative microstates

The segmentation on the chemosensory condition Grand Means for the three groups identified a total of 18 stable topographies, explaining 80.3% of the global variance [Fig. 5(iii), (iv)]. The micro-states, originally named with numbers in crescent order, were renamed with alphabetic letters from a to t to avoid confusion with the maps identified in the mechanosensory segmentation. In detail, the chemosensory stimulus processing was described by eleven micro-states for the control (N), namely a, b, d, e, f, h, i, m, o, s, and t; six microstates for the PM group, namely c, f, g, p, q, and t and by height microstates for the PMA group, namely a, f, c, h, l, n, r, and t. The three groups shared map f from c.a.78 ms to 287 ms, although with notable different duration and onset, and map t from c.a. 636 ms till the end of the epoch. N and PMA groups shared microstate a from the beginning of the epoch to c.a. 46 ms and microstates h from c.a. 236 ms to 441 ms. Finally, PM and PMA groups shared map c that appears in different time frames, at the beginning of the epoch for PM and around 107 ms for PMA. The micro-states analysis clearly reflects the original data, as is visible from Fig. 5(iii), (iv), and (i), where we also highlight the similar topographies with the same color code.

Fitting back to the single subject EEG-grand average. To test the consistency of the group analysis at the single-subject level, we fitted back to the single-subject the shared micro-states among the groups, namely: a, c, f, h, and t, highlighted in Fig. 5(iii). For each micro-state, we extracted the following variables: Duration, GEV, and the first onset within a specific time frame (TF) of interest defined as TF0, within 0 to 223 ms for map a and c, TF1 within 80 to 359 ms, and TF2 361 to 559 ms for map f, TF3, within 639 to 1199 ms for map t and TF4 between 119 and 225 ms, for map h. We additionally extracted the first onset of map f on TF1 and the first onset of map c on TF0.

**Fig. 6:**
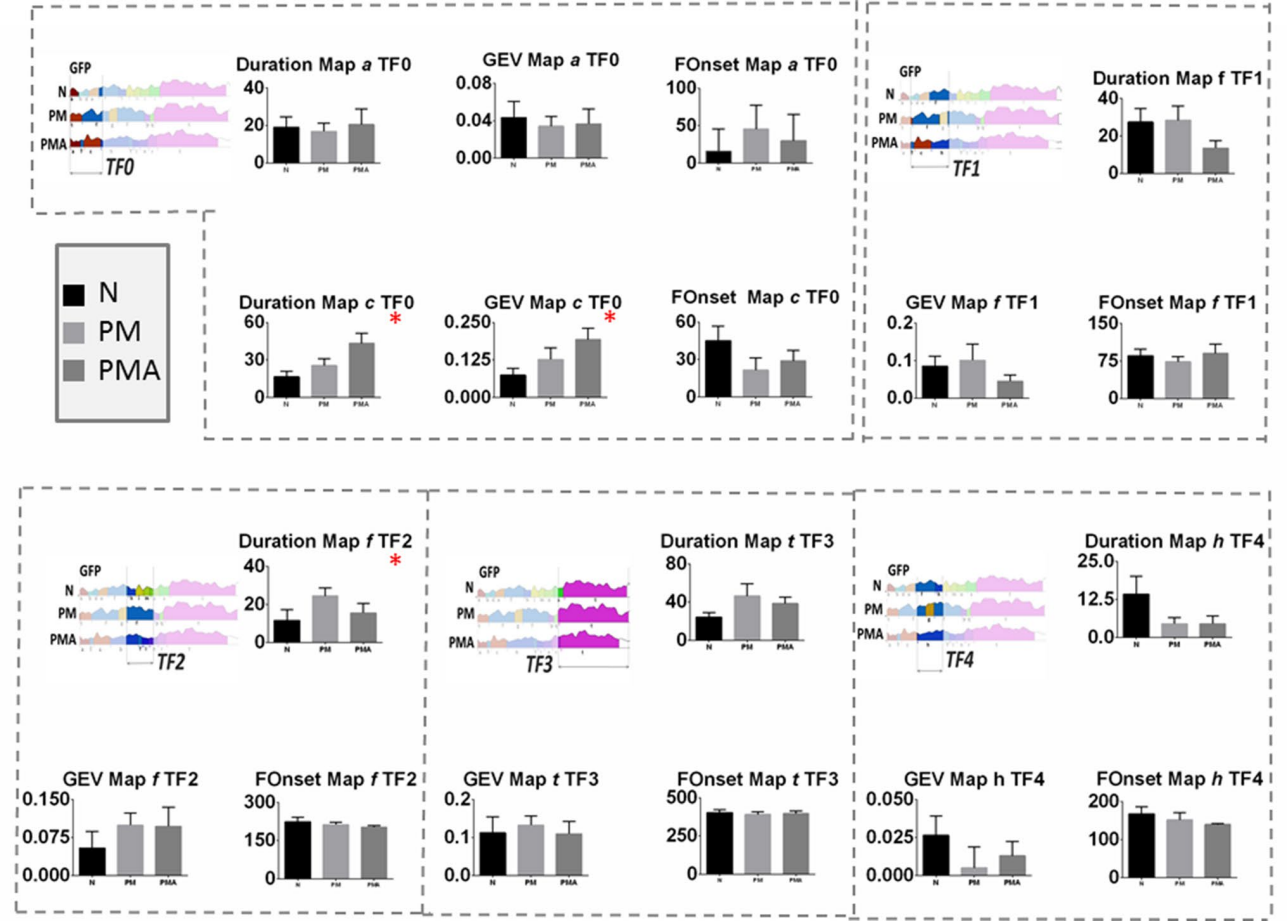
Mean and SE of the variables mean map duration (Duration), global explained variance (GEV) and first onset (FOnset) for map *a,* c, f, t, and h in the time frame (TF) of interest. Statistically significant differences were found form duration and GEV of map *c* within TF0 and map *f* withing TF2. The figure reports also the illustration of the relative TF within the epoch under analysis. TF0: 0 to 223 ms, TF1: 80 to 359 ms, TF2: 361 to 559 ms, TF3: 639 to 1199 ms, and TF4: 119 and 225 ms

A non-parametric Kruskal–Wallis one-way analysis of variance among the three groups was used to test statistical differences on the defined variables. Details of the statistical results are reported in Table 5; mean and SE across the groups are reported in Fig. 6 for each variable. The results showed a significant difference in the mean duration of map c within the TF0. The post hoc test showed that map c was shorter with a smaller GEV for the N-group, confirming the segmentation. Also, map f within the TF2 was shorter with a smaller GEV for the N-group. Figure 6 illustrates the mean values and SE of the tested variables. This suggests that a longer map c in the initial part of the epoch and a longer map f in TF2 indicates a migraine condition.

**Table 5:** Statistical test results for the variables extracted after the fitting back of the segmentation to the single subject grand average for the chemosensory condition

TF	Map	Mean duration	GEV	First onset
		p (H)		
0	a	0.8803 (0.255)	0.8405 (0.348)	–
	c	0.0298 (7.026)*	0.0471 (6.109)*	0.1991 (3.228)
4	h	0.4700 (1.510)	0.6571 (1.134)	–
1	f	0.4675 (1.521)	0.4675 (1.521)	0.8945 (0.223)
2	f	0.0490 (6.032)*	0.0516 (5.927)^a^	–
3	t	0.0655 (5.452)	0.2652 (2.655)	–

	N	PM	PMA	Effect size (r)
Mapc (TF0)				
Mean duration CI(95%)	16.50 (7.01, 26.00)	25.50 (13.98, 37.02)	43.21 (25.60, 60.82)	0.38
GEV CI(95%)	0.074 (0.025, 0.123)	0.127 (0.045, 0.208)	0.193 (0.112, 0.273)	0.34
Mapf (TF2)				
Mean duration CI(95%)	11.64 (− 0.38, 23.66)	24.40 (15.05, 33.76)	15.50 (4.63, 26.37)	0.32

p, p-value of the statistical test, H, the value of the Kruskal–Wallis test. In addition, mean values, 95% confidence intervals [CI(95%)], and effect size of the variable with statistically significant results are reported in detail

**p < 0.005; *p < 0.05

^a^Indicates a value at the threshold supposes a trend of significance

**Fig. 7:**
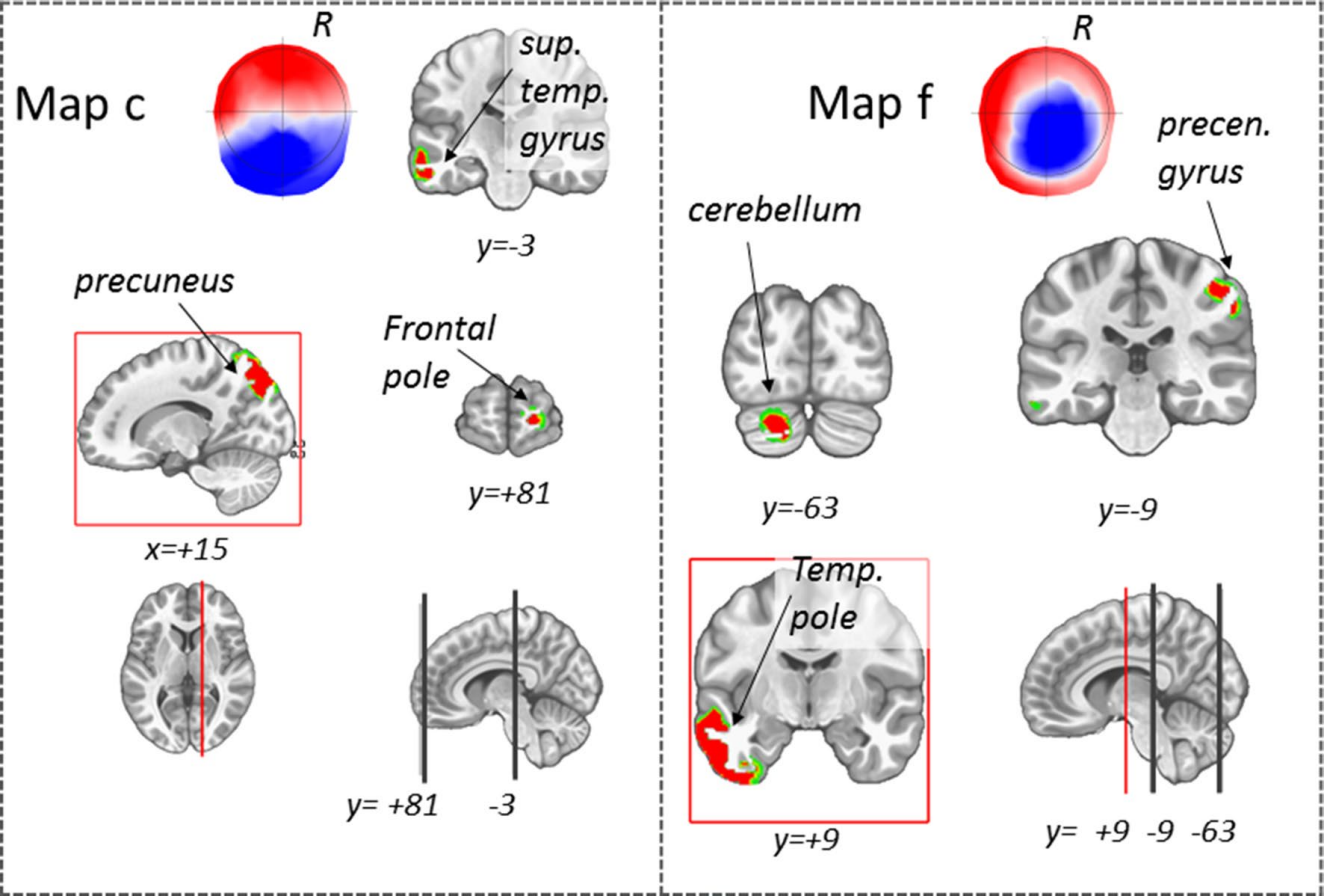
Source localization of the microstate of interest, map c, f within the chemosensory condition. Maximal activities are highlighted within a red frame. R = right, coordinates are reported in MNI standard space

Source analysis for the chemosensory condition: The source analysis of map c revealed activity in the right precuneus, left temporal Mid area, and the frontal superior gyrus. The source analysis of map f indicated activity in the temporal pole mid-left, the cerebellum Crus 1 left, and the precentral gyrus right. Brain areas involved in the maps discussed above are illustrated in Fig. 7.

Table 6 summarizes the maps of interest, the correspondent maximum of brain activities in MNI coordinates, and the relative brain areas nomenclature.

**Table 6:** Source localization of the relevant brain topographies for migraine condition, migraine with aura and control

	Map	MNI (mm)			Brain area		
		x	y	z	Brain lobe	Brain sub-area	Brodnmann area
General migraine	c	15.11	− 57.41	39.28	Parietal lobe	Precuneous R	7
		− 63.45	− 3.02	− 15.11	Temporal lobe	Superior temporal gyrus L	22/21
		15.11	81.58	− 3.02	Frontal lobe	Frontal pole R	10
	f	− 51.36	9.06	− 33.23	Temporal lobe	Temporal pole L	21/20
		− 21.15	− 63.45	− 33.23	Cerebellum	Posterio lobe	Crus 1
		51.36	− 9.06	45.32	Frontal lobe	Precentral gyrus R	4
	12	9.06	− 33.23	− 57.41	Cerebellum	Posterio lobe R	9
		− 51.36	27.19	15.11	Frontal lobe	Inferior frontal gyrus opercular part	45/46
Migraine with aura	7	− 33.23	− 45.32	− 57.41	Cerebellum	Posterior lobe L	8
		45.32	− 45.32	− 45.32	Cerebellum	Posterior lobe R	Crus 2
		3.02	63.45	− 21.15	Frontal lobe	Fusiform gyrus R	11
		− 9.06	69.49	− 21.15	Frontal lobe	Lingual gyrus L	11
	9	15	− 63	− 39	Cerebellum	Posterior lobe L	8
		− 15	− 63	− 39	Cerebellum	Posterior lobe L	8
		− 51.36	15.11	− 39.28	Temporal lobe	Inferior temporal gyrus L	38/20
Control	13	57.41	− 9.06	39.28	Parietal lobe	Postcentral gyrus R	3
		− 45.32	21.15	− 39.28	Temporal lobe	Temporal Pole L	20

#### Correlation Analysis with Clinical Data

##### General Migraine Condition

Our results showed a relationship between the general migraine disorder (with and without aura) and map c and f within the chemosensory condition and map 12 within the mechanosensory condition. To test the relationship between the eCSD signal of the corresponding elicited brain activities and the clinical data available for the PM and PMA groups, namely BDI, MIDAS, HIT-6, ‘Years with migraine’ and ‘migraine days last 3 months’ we performed a Spearman rho correlation analysis. There was a strong positive correlation between eCSD in the right precuneus, left temporal pole, and right cerebellum and the variable ‘Years with migraine’, with respective values r = 0.61, p = 0.039, CI(95%) = (0.03, 0.88); r = 0.60, p = 0.043, CI(95%) = (0.02, 0.88); and r = 0.62, p = 0.34, CI(95%) = (0.05, 0.88) indicating a high level of eCSD associated with longer years of persistent migraine disease.

Further, medium to low correlation have been found for patient reported measures as HIT-6 [left temporal pole: r = − 0.43, p = 0.02, CI(95%) = (− 0.69, − 0.08)]; right cerebellum: r = − 0.37, p = 0.05, CI(95%) = (− 0.65, − 0.01). Since other patient reported measures as Midas did not show any correlation, we view these results with caution, also due to subjectivity and inter-individual variability in a rather small group of patients.

**Fig. 8:**
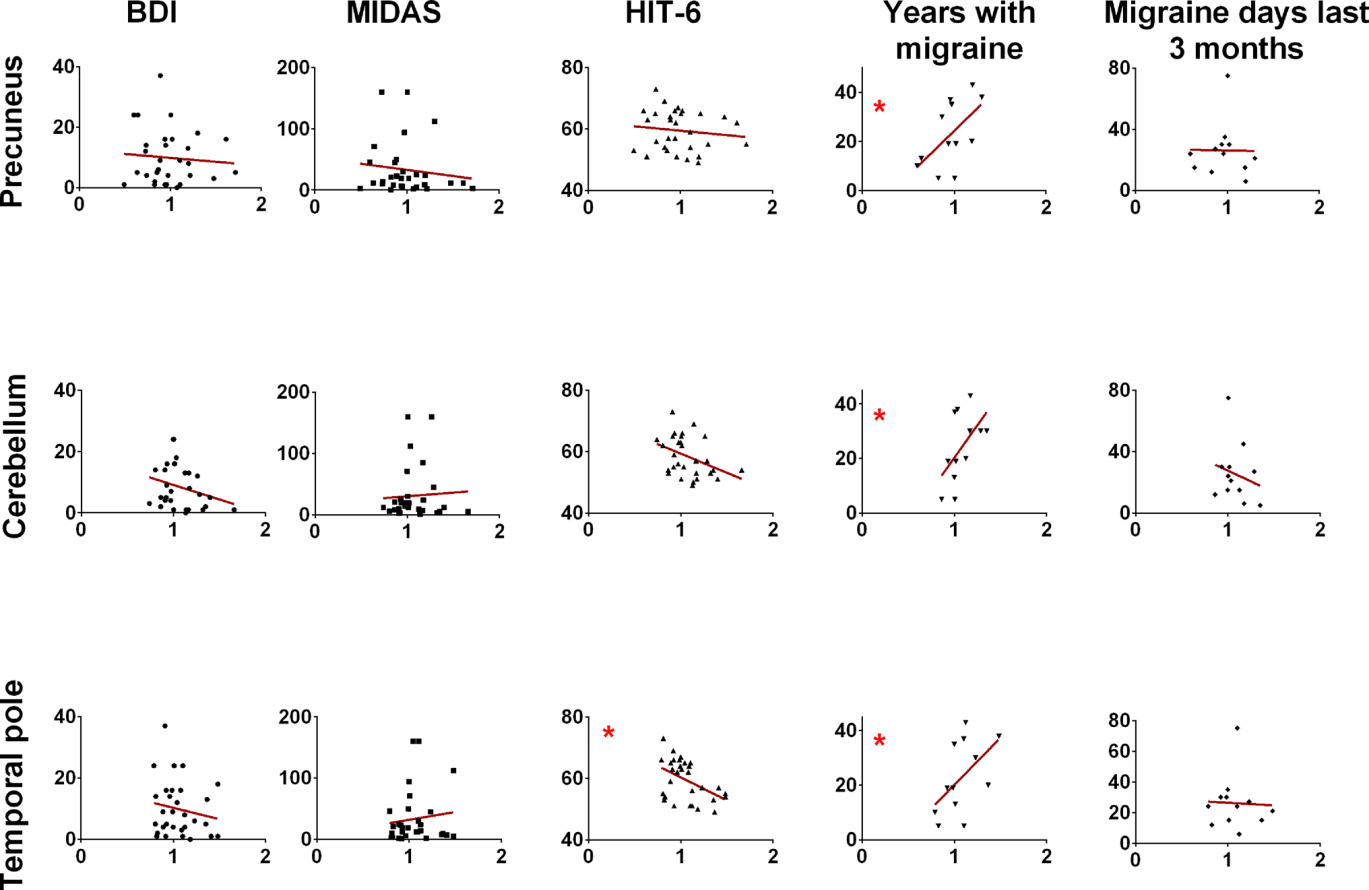
Spearman correlation analysis between the estimated current source density (eCSD, x-axis) and clinical scores. The eCDS was extracted in a ROI centered in the maximum activity in the relative brain region, precuneus, cerebellum and temporal pole, respectively for map c, map f, and map 12. Spatial coordinates of the brain areas are reported in Table 6

Details of the correlations are reported in Fig. 8 and Table 7.

**Table 7:** Spearman rho Correlation analysis and significance between the eCSD for the max brain activity in map c (precuneus), f (temporal pole) and 12 (cerebellum) and the clinical scores BDI, MIDAS, HIT-6, Years with migraine, Migraine days last three months

	Spearman r	BDI	MIDAS	HIT-6	Years with migraine	Migraine days last 3 months
Precuneus	r (p)	− 0.03 (.88)	− 0.15(.44)	− 0.17 (.38*)*	0.61 (.04)*	− 0.03 (.91*)*
	Effect size^$^ (CI)				Strong (.032, .880)	
Temporal Pole	r (p)	− 0.16 (.41)	0.11 (*.55)*	− 0.43 (0.02)*	0.60 (0.04)*	− 0.18 (.57*)*
	Effect size^$^ (CI)			Moderate (− .693, − .076)	Relatively strong (021, .878)	
Cerebellum	r (p)	− 0.22 (.26)	0.01 (.94)	− 0.36 (.05)	0.62 (.03)*	− 0.21 (.051)
	Effect size^$^ (CI)				Strong (.055, .886)	

*p < .05

^$^as defined in Rea and Parker (1992)

##### Migraine Condition with Aura

Segmentation analysis and the fitting back to the single subjects of the variable defining the microstates have indicated map 7 and the strength and duration of map 9 as specific topographies for the migraine condition with aura. To confirm these results, we have computed the results of the inverse solution and the related eCSD in the sources for each group. A Kruskal–Wallis test revealed a statistically significant difference in eCSD signal extracted from the ROI with maximum brain activity sources of map 9 (right cerebellar cortex) across the three different groups, N [eCSDmean = 0.93, CI(95%) = (0.88, 0.99)], PM [eCSDmean = 0.92, CI(95%) = (0.86, 0.98)], and PMA (eCSDmean = 1.16, CI(95%) = (1.02, 1.31), H = 8.496, p = 0.0143, effect size (r) = 0.47. This confirms an increase in the strength of the eCSD signal in the right cerebellar cortex during the tf1 for the PMA group.

**Fig. 9:**
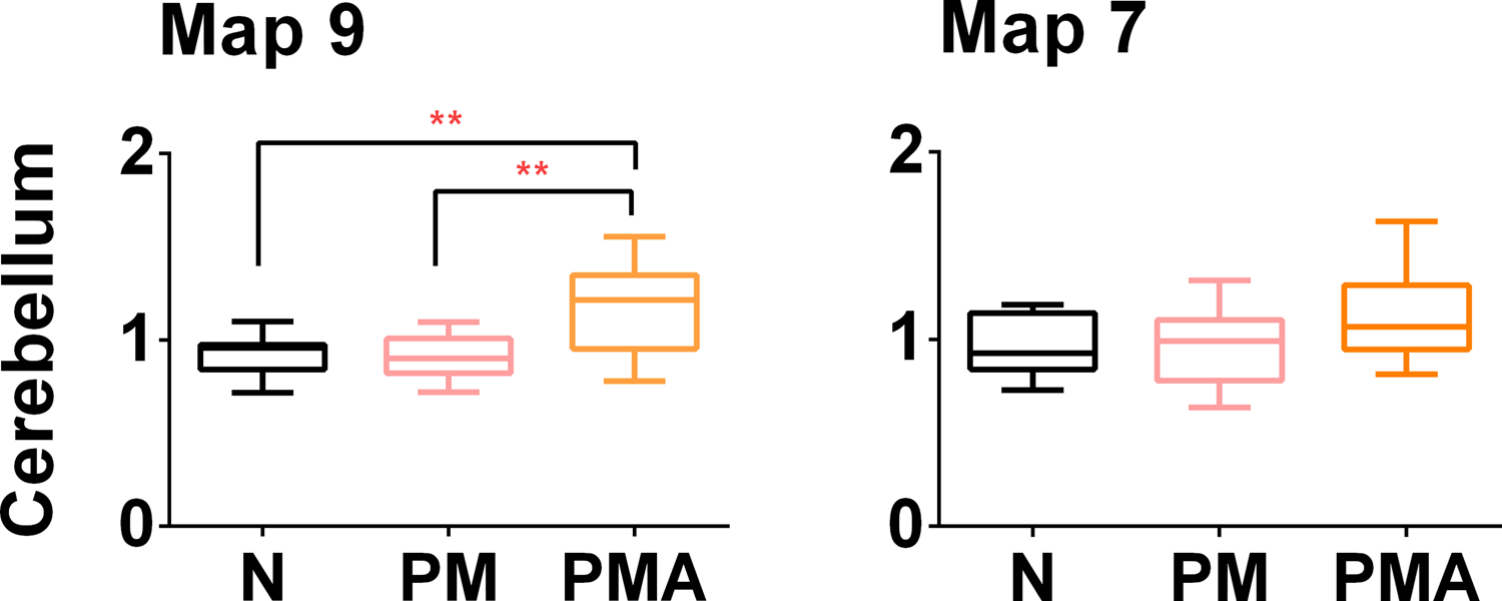
Box plots of eCSD (y-axis) extracted in a ROI centered in the maximum activity of the cerebellum, for map 9 and map 7. Spatial coordinates of the cerebellar areas are reported in Table 6. N, control; PM, patients with migraine without aura; PMA, patients with migraine with aura. Significant differences are indicated with *

Data are illustrated in the box plot of Fig. 9.

## Discussion

Our study revealed characteristic EEG topographies for migraine patients with or without aura and healthy controls. Map c, map f, in the chemosensory condition (Figs. 5, 6) and map 12 (mechanosensory condition, Figs. 2 and 3) in the late part of the epoch (tf4) were specific for the general migraine conditions (with and without aura). The fit corroborated the identification to the single-subject data that, coherently with the group analysis, explained in a statistically significant way the data variance. The relative source analysis indicated a maximum of activities in brain areas respectively located in the precuneus right (BA7), temporal pole left (BA21/22), and the cerebellum in the right posterior lobe (area IX) (Figs. 4 and 7). eCSDs, extracted in the ROI centered in the maximum activity of the previously mentioned brain areas, positively correlated with the clinical score ‘years of migraine.’ (Fig. 8, Table7) This indicates an intensification of brain response in the above-mentioned areas with the duration of migraine.

Evidence also suggested that modulation in strength and duration of the signal for map 9 in tf1 and the relative eCSD in the left posterior lobe of the cerebellum (area VIII) is specific to migraine with aura condition.

Regarding the clinical and psychophysical data, there were no differences between the study groups in olfactory identification ability. Trigeminal sensitivity however, was significantly higher in migraine patients with and without aura than controls. The BDI score was significantly higher in the PMA groups (but a trend was evident also for the PM group), indicating more depression-relevant symptoms in migraine patients than controls. Anxiety and depressive disorder are known comorbidities in migraines resulting in an additional disease burden (Alwhaibi and Alhawassi 2020). Interestingly, tracking performance was significantly better in the PMA group then control in all the conditions. A similar trend also appeared between PMA and PM group. Expectation, vigilance, and reappraisal modulate the ability to perceive pain (Keltner et al. 2006; Meyer 2002). We believe that the trigeminal hypersensitivity, and the increased performance in the migraine with aura group in our study, is an index of this enhanced cognition of pain.

### EEG Identification of General Migraine Condition

In our study, we were able to identify maps and relative brain areas related to the migraine condition.

The *precuneus* is part of the default mode network (DMN) and is involved in pain sensitivity (Goffaux et al. 2014; Emerson et al. 2014; Schwedt et al. 2015a, b) and modulation of pain in both acute and chronic conditions (Emerson et al. 2014) and specifically in chronic migraine (Schwedt et al. 2015a, b), involving overactivity and abnormal linkage with cortical networks (Zhang et al. 2016; Tomasi and Volkow et al. 2011; Huang et al. 2011; Griebe et al. 2014; Schwedt et al. 2015a, b; Goffaux et al. 2014). In a work that studied the functional connectivity (FC) of the precuneus in migraines, the authors observed an abnormal FC wihin the DMN (Zhang et al. 2016), suggesting this area playing a crucial role in dysfunctions commonly associated with migraine. Our data showed that: (1) precuneus was activated during map c within the chemosensory stimulation, (2) map c was only present in the migraineurs group, with and without aura, and (3) the result was statistically confirmed at the single-subject level. Moreover, the eCSD from the ROI centered in the precuneus maximum activity was positively correlated with the variable ‘years with migraine’, confirming the crucial role of this specific area in the pathophysiology of the migraine.

The *temporal pole* (TP) participates in pain processing by mediating affective responses to painful stimuli and is a multisensory convergence zone responsible for processing visual, olfactory, and auditory stimuli (Demarquay et al. 2008; Moulton et al. 2011). The activity that we observed in this area is positively correlated with the duration of migraine. Our result agrees with the TP hyperexcitability found by Moulton et al. (Moulton et al. 2011) in interictal migraine patients. The same area was found exacerbated during migraine attacks (Burstein et al. 2010). Notably, in agreement with our results, the temporal pole has been shown to have atypical activation and atypical functional connectivity in several migraine fMRI studies (Maleki et al. 2012a, 2012b; Tessitore et al. 2013; Stankewitz et al. 2011). Altogether this indicates that the trigeminal response in the temporal pole can differentiate migraine brain from healthy controls, and it can possibly have a role in migraine etiology.

The *cerebellum* has been linked to processing sensorimotor, cognitive, and affective information of pain-perception (Moulton et al. 2011; Mehnert and May 2019; Ruscheweyh et al. 2014) and pain in migraines (Carpenter and Hanna 1961; Coombes and Misra 2016; Kros et al. 2018; Mehnert and May 2019; Vincent and Hadjikhani 2007). A recent study has explored the alteration of gray matter volume and diffusion properties of the cerebellum in migraineurs. (Qin et al. 2019). Hyperactivity in the cerebellum in migraine patients in response to negative stimuli, similar to our chemosensory condition, has been linked to decreased inhibition of aversive processing that can act as trigger to aggravate headache in migraine patients (Wang et al. 2017).

### EEG Changes in Migraine Patients with Aura

The two subgroups of the disease, migraine with and without aura, are thought to differ in terms of pathophysiology (Zhang et al. 2016). Our results have shown an increase of duration in time and strength of map9 in tf1 during the mechanosensory stimulation for the PMA group and a significant increase of the eCSD localized in the posterior lobe cerebellum area VIII (Figs. 3 and 6, Table 5). This is in line with the reported evidence for interictal cortical hyperexcitability in migraine, which is most pronounced in patients with aura (Brigo et al. 2012). The characteristic modulation of the signal, individuated by the modulation in strength and duration of map 9, represent a signature of EEG-noxious response of the migraine with aura condition.

Up to now, several studies have linked clinical and pathophysiological evidence between general migraine and lesions in the cerebellum (Kruit et al. 2004, 2005, 2006). Our results on the increase of signal in the cerebellar cortex in migraine and especially in the PMA group corroborate previous works with the addition that we identified the areas (location and time tf4 after stimulus onset) in the cerebellum posterior lobe area IX which positively correlated with the duration of the symptomology for both migraine and migraine with aura.

### Altered Sensory Processing in Migraine

Different maps and relatively different brain areas are highlighted with different nociceptive trigeminal input, possibly due to the different valence of the stimuli. This agrees with previous studies indicating the brains of migraineurs might also be interictally different in terms of how they respond to sensory stimulation (Boulloche et al. 2010; Charles 2013; Coppola et al. 2007; Russo et al. 2012a, b; Liu et al. 2012) suggesting a permanent interictal alteration of the function and structures of the brain as a consequence of the migraine state.

To the best of our knowledge, our study is the first one involving a chemosensory trigeminal stimulation of patients in an interictal condition.

The air-puff condition can be compared to other cutaneous noxious stimuli. Several works have reported a hypersensitivity to touch, indicated as mechanical allodynia. This response is typical of up to two-thirds of migraine patients (Lipton et al. 2008; Selby and Lance 1960) especially recognized in the cephalic/facial region and registered at the cerebral level using fMRI techniques. Our experiment observed an increase in the duration and intensity of the EEG signal for the PMA group within the air-puff condition in the temporal segment tf1 where map 9 was present (Fig. 2). The increase of duration and intensity of map 9, expressed by the GEV, were statistically significant also at the single-subject level. This can be explained by hyperactivity typically associated with migraines with aura. We also added the information that the relative hyperexcited brain area has its maximum in an area of the right cerebellum, area 8 (Fig. 9a), with other local maxima in the left cerebellum and the inferior temporal gyrus.

Pain processing, such as pain memory and prior pain experience, affects neurocognitive aspects of the human brain (Wiech et al. 2008). We believe that the trigeminal hypersensitivity, and the increased performance, especially in the migraine with aura group in our study, is an index of this enhanced cognition of pain. Similar explanation can be given to the activity in the motor cortex (BA4) and cerebellum due to the preparation of nocifensive reflexes initiated by the hypersensitivity to the trigeminal-stimulation (Ruehle et al. 2006), and to movement suppression or movement evoked by the noxious stimulus itself (Davis et al. 2002).

Finallythe results of this work show also that a simple mechanosensory/chemosensory experimental paradigm targeting the trigeminal nerve might pinpoint topographies and relative brain areas discriminating migraineurs from controls. This result is significant since migraine is not commonly associated with a structural abnormality detectable with standard MRI or CT procedure (Evans et al. 2020).

## Supplementary Information

Below is the link to the electronic supplementary material.

Supplementary file1 (DOCX 192 KB)

## Data Availability

The datasets generated during and/or analysed during the current study are available from the corresponding author on reasonable request.
