# Supplementary material for: Migraine Type-Dependent Patterns of Brain Activation After Facial and Intranasal Trigeminal Stimulation
Source: Brain Topogr. 2022 Nov 12;36(1):52–71. doi: 10.1007/s10548-022-00924-x (PMC9834371; doi:10.1007/s10548-022-00924-x)

Manuscript title:

**"Migraine type-dependent patterns of brain activation after facial and intranasal trigeminal stimulation"**

Corresponding author: Emilia Iannilli

Email: emilia.iannilli@googlemail.com

**Supplementary Information**

- **Pilot study for intensity stimulus matching**

Fifteen individuals were exposed to chemical, electrical, and mechanical conditions. They were asked to evaluate the conditions on a scale from 0 (not perceived) to 100 (extremely strong) in intensity. Initially, the chemical condition were split into two levels; CO2_low (44% v/v CO2) and CO2_high (56% v/v CO2). As regards the electrical stimulation, we first set the electrical threshold (minimum level of electrical stimulus detection), then increased the relative experimental condition by 20% of this value. The mean ratings and standard deviation for the group in each condition are reported in SI-table 1.

SI-Table 1. Intensity ratings of the various stimuli: Fifteen subjects evaluated intensity in a pilot study for the different stimuli conditions.

| stimulation | Mean (a.u.)  Intensity | s.d. (a.u.) |
| --- | --- | --- |
| CO2_low | 33.67 | 6.18 |
| CO2_high | 54.60 | 14.51 |
| Electrical | 37.07 | 10.73 |
| Puff | 39.07 | 13.24 |

A Friedman test revealed a statistically significant difference across the 4 intensity ratings (χ^2^(3, n=15) =20.3, p<0.001). Higher median (Md) intensity values were observed for CO2_high (Md=52) compared to CO2_low (Md=31), electrical condition (Md=32), and mechanical condition (Md=33). A posthoc test (Wilcoxon) with Bonferroni correction showed no significant differences between CO2_low vs. electrical condition nor for CO2_low vs. mechanical or electrical condition vs. Puff (p>0.16). As the final set of experimental conditions, we chose 1) cutaneous mechanical stimulation using air puffs, 2) cutaneous electrical stimulation (20% higher than the individual threshold), 3) intranasal chemical stimulation with gaseous CO2 (44% v/v CO2).

- **Extensive Clinical data**

Of 17 patients with migraine with aura, 2 showed pronounced migraine prodromes, only one was on prophylactic migraine medication and 2 on non-medication prophylactic therapy. Four of the patients complained about tension type headache or facial pain, and 3 patients about pain in other body regions. Six patients were on prescribed acute migraine medication, and 1 consumed analgesic for pain conditions other than migraine. A psychological diagnosis was seen in 4, and concomitant somatic diseases in 4 patients (SI-Table2).

SI-Table2. Full clinical information of episodic migraine patients with aura

| Age | **Sex** | **BDI** | **Aura** | **Midas** | **HIT-6** | **years with migraine** | **migraine days last 3 month** | **migraine prodromes** | **acute/prophylactic migraine medication** | **prophylactic medication** | **nonmedication prophylaxis** | **other headache or facial pain** | **other pain locations** | **analgesics** | **psychological diagnosis** | **ataractics** | **concomitant diseases** | **other medication** |
| --- | --- | --- | --- | --- | --- | --- | --- | --- | --- | --- | --- | --- | --- | --- | --- | --- | --- | --- |
| 32 | F | 37 | y | 23 | 63 |  |  |  |  |  |  |  |  |  |  |  |  |  |
| 18 | F | 24 | seeing stars, silverish flickery scotoma central, concentric visual field narrowing, hypaesthesia arm, leg, aphasia | 46 | 66 | 10 | 24 |  | Rizatriptane 10 | Metoprolole 47,5 |  |  |  |  | depression,  sociophobia |  | neurodermitis |  |
| 28 | F | 24 | flickery lights temporal, dysesthesia arm | 160 | 65 | 5 | 75 | visual aura hrs. before headache | Ibuprofene 600 |  | Yoga | TTH | low back |  | sociophobia, depression, personality disorder , dependent/ impulsive type |  | asthma, skoliosis |  |
| 58 | F | 18 | y | 112 | 55 | 38 | 21 |  | Almograne 5 |  |  | TTH, Hx of trigeminal neuralgia | chronic widespread pain |  | depression, somatofom pain disorder |  | cataract senilis, D.m. | Metoprolol, Omeprazole, Colecalciferol, L-Thyroxine |
| 51 | F | 24 | flickery scotoma temporal | 71 | 63 | 13 | 15 |  | Zolmitriptane 5 |  |  | TTH |  |  | anorexia nervosa, depression | Citaloprame 10 | OP epidermoid, cyst temporoparietal |  |
| 24 | F | 1 | y | 1 | 69 |  |  |  | Novaminsulfone 500 |  |  |  |  |  |  |  |  |  |
| 27 | F | 2 | dysesthesia arm, leg | 21 | 54 | 5 | 12 | food craving, dizziness | Sumatriptane 50 |  | Sports | TTH | low back | Ibuprofene |  |  |  |  |
| 28 | f | 1 | y | 7 | 57 |  |  |  |  |  |  |  |  |  |  |  |  |  |
| 39 | F | 16 | y | 12 | 62 |  |  |  |  |  |  |  |  |  |  |  |  |  |
| 31 | F | 12 | y | 160 | 65 |  |  |  |  |  |  |  |  |  |  |  |  |  |
| 26 | M | 1 | y | 3 | 53 |  |  |  |  |  |  |  |  |  |  |  |  |  |
| 26 | F | 5 | y | 3 | 55 |  |  |  |  |  |  |  |  |  |  |  |  |  |
| 22 | F | 5 | y | 8 | 53 |  |  |  |  |  |  |  |  |  |  |  |  |  |
| 24 | F | 1 | y | 5 | 54 |  |  |  |  |  |  |  |  |  |  |  |  |  |
| 26 | F | 0 | y | 5 | 56 |  |  |  |  |  |  |  |  |  |  |  |  |  |
| 27 | M | 7 | y | 19 | 63 |  |  |  |  |  |  |  |  |  |  |  |  |  |
| 25 | F | 9 | y | 50 | 64 |  |  |  |  |  |  |  |  |  |  |  |  |  |
| Age | **Sex** | **BDI** | **Aura** | **Midas** | **HIT-6** | **years with migraine** | **migraine days last 3 month** | **migraine prodromes** | **acute/prophylactic migraine medication** | **prophylactic medication** | **nonmedication prophylaxis** | **other headache or facial pain** | **other pain locations** | **analgesics** | **psychological diagnosis** | **ataractics** | **concomitant diseases** | **other medication** |

Of 17 patients with migraine without aura, 5 showed pronounced migraine prodromes, 4 were on prophylactic migraine medication, and 9 on nonmedication prophylactic therapy. In addition, 6 of the patients complained about tension type headache or facial pain, and 8 patients about pain in other body regions. 11 patients took acute migraine medication and 6 consumed analgesics for pain conditions other than migraine. A psychological diagnosis was seen in 3; concomitant somatic diseases in 4 patients. (SI-Table 3)

SI -Table3. Full clinical information of episodic migraine patients without aura

| **Age** | **Sex** | **BDI** | **Midas** | **HIT-6** | **years with migraine** | **migraine days last 3 month** | **migraine prodromes** | **acute migraine medication** | **prophylactic medication** | **nonmedication prophylaxis** | **other headache or facial pain** | **other pain locations** | **analgesics** | **psychological diagnosis** | **ataractics** | **concomitant diseases** | **other medication** |
| --- | --- | --- | --- | --- | --- | --- | --- | --- | --- | --- | --- | --- | --- | --- | --- | --- | --- |
| 26 | F | 3 | 12 | 64 | 10 | 15 |  | Sumatriptane 100, Naproxene 500 | Mg 300 | BF | TTH |  | n |  |  |  |  |
| 43 | F | 13 | 70 | 61 | 20 | 6 |  | Zolmitriptane 5 |  | Sports | PIFP, TTH, CMD | low back, Joints | Ibuprofene | depression, somatoform disorder |  | rheumatoid arthritis, hyperthyreosis | Methotrexat |
| 50 | F | 13 | 85 | 53 | 30 | 45 | dizziness | ASS 500 | Amitriptyline 25 |  |  | low back, feet | Etoricoxibe | depression, anxiety disorder | Amitriptyline 25 | psoriasis, spondylarthrosis | Pregabaline 50 |
| 54 | F | 2 | 5 | 54 | 30 | 5 | food craving | Almotriptane 5 |  | PMR |  | low back, shoulder | Ibuprofene, Novaminsulfone |  |  | skoliosis, aHTN |  |
| 57 | F | 4 | 94 | 67 | 35 | 35 | food craving, emotional instability | Sumatriptane 50 |  | Sports | TTH | low back, ellbow |  |  |  |  |  |
| 23 | F | 4 | 2 | 65 |  |  |  |  |  |  |  |  |  |  |  |  |  |
| 21 | F | 9 | 25 | 59 |  |  |  |  |  |  |  |  |  |  |  |  |  |
| 26 | M | 0 | 2 | 50 |  |  |  |  |  |  |  |  |  |  |  |  |  |
| 55 | F | 8 | 24 | 51 | 43 | 6 |  | Rizatriptane 5 |  | Yoga | TTH | shoulder | Ibuprofene |  |  |  |  |
| 55 | F | 6 | 45 | 57 | 30 | 27 | yawing | Rizatriptan 10 , Naproxene 500 | Mg 300 | Yoga | TTH, CMD |  |  |  |  |  |  |
| 44 | M | 14 | 6 | 62 |  |  |  |  |  |  |  |  |  |  |  |  |  |
| 50 | F | 5 | 12 | 51 |  |  |  |  |  |  |  |  |  |  |  |  |  |
| 24 | M | 4 | 14 | 56 |  |  |  |  |  |  |  |  |  |  |  |  |  |
| 46 | F | 1 | 30 | 51 | 37 | 24 |  | Rizatriptane 5 | Topiramate 100 | PMR |  | neck |  | agoraphobia, posttraumatic, stress disorder |  | D.m., renal insufficiency |  |
| 40 | F | 13 | 9 | 49 | 20 | 15 |  | Ibuprofen 400 |  | Gymnastics | TTH | low back neck | Ibuprofene |  |  |  | Panoprazole 20 |
| 43 | F | 16 | 19 | 66 | 19 | 30 | food craving | Rizatriptane 10 |  | Breath relaxation |  | shoulder | Novaminsulfone |  |  |  |  |
| 25 | F | 14 | 10 | 73 |  |  |  |  |  |  |  |  |  |  |  |  |  |
| **Age** | **Sex** | **BDI** | **Midas** | **HIT-6** | **years with migraine** | **migraine days last 3 month** | **migraine prodromes** | **acute migraine medication** | **prophylactic medication** | **nonmedication prophylaxis** | **other headache or facial pain** | **other pain locations** | **analgesics** | **psychological diagnosis** | **ataractics** | **concomitant diseases** | **other medication** |

**SI-Figure 1. Grand Mean butterfly plot for the three groups in the study and the two stimulus conditions.** Butterfly plots of 128-channels Grand Average of the chemo -(CO2) and mechano- (PUFF) sensory evoked potentials evaluated for the control group (N), patients with migraine without aura (PM), and patients with migraine with aura (PMA). The recording scale is reported in microvolts on a total time range of 1.2 s.


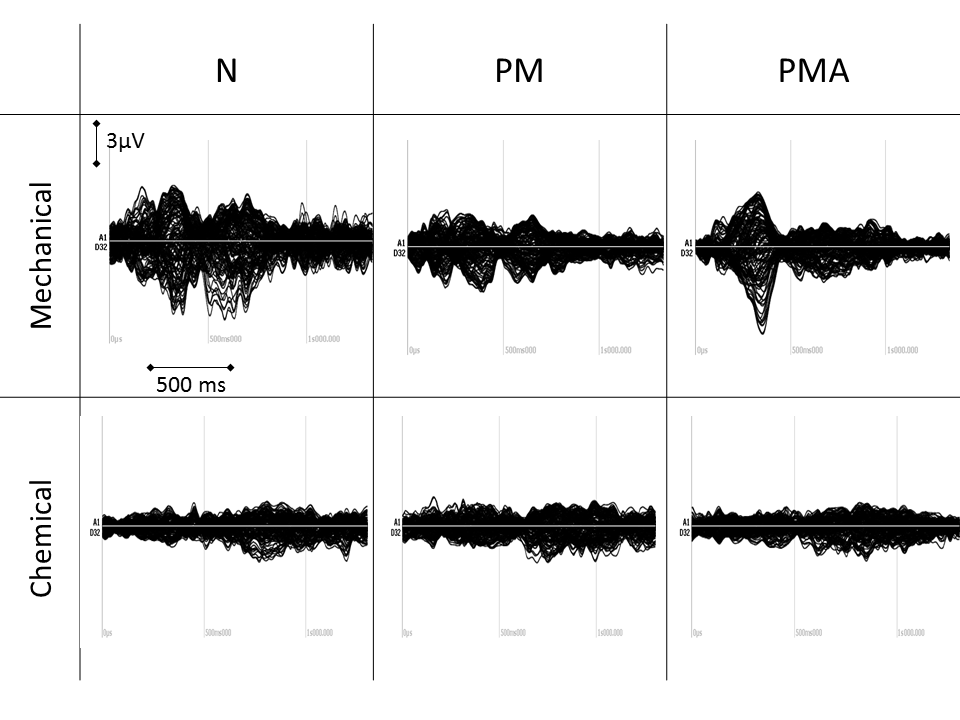

Supplement: Supplementary file 1 — Supplementary file1 (DOCX 192 KB) [file 10548_2022_924_MOESM1_ESM.docx]
